# Values of Selected Strength Parameters of Miscanthus × Giganteus Stalk Depending on Water Content and Internode Number

**DOI:** 10.3390/ma15041480

**Published:** 2022-02-16

**Authors:** Sławomir Francik, Paweł Knapik, Bogusława Łapczyńska-Kordon, Renata Francik, Zbigniew Ślipek

**Affiliations:** 1 Department of Mechanical Engineering and Agrophysics, Faculty of Production Engineering and Energetics, University of Agriculture in Krakow, Balicka 120, 30-149 Krakow, Poland; p.knapik1@wp.pl (P.K.); boguslawa.lapczynska-kordon@urk.edu.pl (B.Ł.-K.); zbigniew.slipek@urk.edu.pl (Z.Ś.); 2 Department of Bioorganic Chemistry, Chair of Organic Chemistry, Jagiellonian University Medical College, 30-688 Krakow, Poland; renata.francik@uj.edu.pl; 3 State Higher Vocational School, Institute of Health, Staszica 1, 33-300 Nowy Sącz, Poland; 4 State Higher Vocational School, Technical Institute, Staszica 1, 33-300 Nowy Sącz, Poland

**Keywords:** lignocellulosic biomass, miscanthus stem, modulus of elasticity, maximum stress, bending test, compression test

## Abstract

So far, there are no results for research on the biomechanical parameters of giant miscanthus stalks taking into account both the influence of moisture content and the internode, from which the samples were taken. Therefore, the aim of the research was to comprehensively investigate the influence of the internode number (NrNod) and water content (MC) on the values of selected biomechanical parameters (modulus of elasticity and maximum stress) determined using various stress tests (three-point bending and compression along the fibers). The research was carried out for dry stalks of different humidities and for different internodes. The results obtained in this study proved that the independent variables of the water content and the internode number cause a statistically significant influence on the values of the examined biomechanical parameters of the miscanthus stem: the modulus of elasticity in compression, the maximum stress in compression, the modulus of elasticity in bending and the maximum stress in bending. The values of the modulus of elasticity (MOE) increase when increasing the NrNod. For individual internodes, MOE values are higher with a higher MC. The values of the maximum stress (σ) also increase when increasing the internode number. For individual internodes, the σ values are lower with a higher MC.

## 1. Introduction

The requirements currently imposed on the industry related to the goals of sustainable development and regarding the reduction of the negative impact on the environment, limiting the consumption of non-renewable raw materials, minimizing non-biodegradable waste make biomass a key raw material for various applications [1]. Scientific research mainly concerns the use of lignocellulosic biomass, which consists mainly of cellulose, hemicellulose and lignin. Lignocellulosic biomass is a source of both energy and carbon [2].

One of the very important applications of plant (lignocellulosic) biomass is its use as a renewable energy source that can be a sustainable alternative to fossil fuels [3]. Biomass can also be used in the sustainable production of liquid fuels and high value-added chemicals [4]. The percentage share of cellulose, hemicellulose and lignin in a given type of biomass may vary, which significantly affects its energy parameters and the possibility of using it for the production of biofuels [5]. Depending on the origin of the biomass (classification according to ISO 17225-1:2014 [6]), research is carried out on woody [7,8,9], and herbaceous biomass [10,11], but also pomaces, fruit stones, kernel shells and other residuals [12,13,14,15], and recently on so-called water biomass, or algae, for example, hyacinths [16,17].

The conducted scientific research relates largely to energy applications of biomass (e.g., combustion or gasification), recovery of platform chemicals and the production of char, a carbon-rich solid material [2]. Biochar has been used for soil amending and site restoration, energy storage, catalysis and electrochemistry, as well as for the production of highly porous activated biochar for processes in which adsorption plays a major role (e.g., for water treatment) [2,18,19]. Research is also ongoing to develop 3D printable biopolymer composites with improved performance [20]. An interesting research topic is the use of lignocellulosic biomass-derived carbon in flexible supercapacitors used in smart textiles, which can perform a number of functions in various fields, such as health monitoring, medical care, sports, military [21].

The giant miscanthus (Miscanthus × Giganteus, perennial C4 grass, sterile miscanthus sinensis and miscanthus sacchariflorus hybrid) is one of the most productive plant species that, thanks to the fast and abundant biomass production, can provide the required amounts of the raw material. It is characterized by a high biomass production potential, which means that it is used to establish industrial biomass plantations throughout Europe and the USA [19,22,23,24,25,26]. Average giant miscanthus yields range from 5 to 55 Mg ha^−1^, which makes it one of the most productive terrestrial plants in a temperate climate [3]. The amount of biomass yield from miscanthus crops is directly influenced by the C4 photosynthesis cycle, which is characterized by increased efficiency and faster biomass production compared to the C3 plants. Additionally, the high productivity of biomass, which is characterized by miscanthus, increases the plant’s absorption of the greenhouse gas, which is CO_2_ [27]. Miscanthus also has a high water uptake efficiency [28].

Giant miscanthus can be successfully used in the production of compact solid biofuels [29,30,31,32], biogas and bioethanol [33,34,35]. Miscanthus’ biomass is used in the production of technical papers, cardboard, packaging plywood or fibers for lining packaging, it can also be a substitute for plastics in the production of packaging [36]. Studies are carried out on Miscanthus fibers to develop renewable construction materials—lightweight concrete [37]. Miscanthus × Giganteus can also be a source for cellulose nanocrystals to PVAc (poly(vinyl acetate)) nanocomposites [38].

Determining the mechanical properties of materials of plant origin is necessary due to their extraction and further processing. In order to correctly design the structures of machines intended for processing a given plant, it is necessary to know the load of working elements, which depends on the mechanical properties of the processed material (plant). In the case of miscanthus, it is first necessary to harvest mechanically (cutting the stalks), then there are various biomass processing processes (shredding, pressing, etc.). In addition, biomass must undergo transport processes many times (including loading and unloading) [3,39,40,41,42,43,44,45,46]. Biomechanical properties can also be used as a selection criterion in a plant breeding program—assessing and adjusting stem mechanical traits may improve stem lodging resistance [47].

Typical laboratory tests aimed at determining the biomechanical properties of plant stems include:

- Bending tests: carried out on, e.g., miscanthus stalks, Arundo donax reed stalks and Gramineae reed stalks, switchgrass, vetiver, tall fescue, sainfolin or sorghum stems [40,42,44,47,48,49,50,51,52,53,54];
- Compression tests along or across the grain: carried out on Gramineae reed stalks, giant reed Arundo donax stalks, miscanthus, wheat stems, barley stems and corn stover [50,51,52,55];
- Tensile tests: carried out on giant miscanthus stalks, Arundo donax reed stalks, Gramineae reed stalks, wheat, corn, barley and patchouli plant [40,43,50,51,52];
- Shearing tests: carried out on miscanthus stalks, Gramineae reed stalks [40,44,51];
- Cutting tests: in which cutting energy and specific cutting force of, for example, miscanthus or sainfolin, were determined [3,40,41,42];
- Impact strength test: carried out on giant miscanthus [44].

Water content is one of the basic factors influencing the physical and mechanical parameters of plants. However, only a few studies have analyzed the effect of water content on the biomechanical [54] properties of plant stems [42,56].

Mechanical properties also differ depending on the growth stages (vegetative stems; green internodes, dry internodes) [54]. Another factor that significantly influences the values of biomechanical parameters of plants from the grass family Poaceae (in this case miscanthus) is the internode from which the samples for strength tests are taken. Different internodes of the same stalk have different values of biomechanical parameters [40,44,48,49,57,58,59,60,61].

An additional difficulty in unequivocally determining the values of miscanthus biomechanical parameters is the large variation between plants. Even the stalks of the same field differ not only in dimensions but also in the values of strength parameters. There is much greater variation between plants cultivated in different locations—the influence of different soil conditions, fertilization, or environmental conditions (groundwater level, rainfall, temperature or sunlight).

In the case of plant materials, the results of measurements of biomechanical parameters are also influenced by the type of strength test and the way it is carried out, including the assumed geometric dimensions of the samples as well as their grain orientations [52,53,62,63,64,65]. In the case of plants from the grass family, the transverse dimensions of the samples are most often determined by the dimensions of the stem (the experiments are carried out on the full cross-section of the stem), so it is difficult to study the influence of the sample size. Only in the case of strength tests of bamboo shoots with large stem diameters, there are many tests on samples cut from the side of the stem.

It was also observed that the maturing of the plant material has an impact on the values of biomechanical parameters. Research on how it affects the strength values (compressive strength, horizontal shear strength, modulus of rupture and modulus of elasticity) was conducted, for example, for bamboo [59,66].

The research aimed at determining the biomechanical parameters of the giant miscanthus was conducted by several research teams. These were primarily bending tests in which mainly the modulus of elasticity was determined (Table 1). The obtained MOE values vary considerably. Therefore, in this paper, an attempt was made to explain such a large diversity of research results.

So far, there are no results of research on the biomechanical parameters of giant miscanthus stalks taking into account both the influence of moisture content and the internode from which the samples were taken. Only a few research teams independently analyzed MOE bend changes depending on the internode number (Table 1).

Therefore, the aim of the research was to comprehensively investigate the influence of the internode number (NrNod) and water content (MC) on the values of selected biomechanical parameters (modulus of elasticity and maximum stress) determined using various stress tests (three-point bending and compression along the fibers). The research was carried out for dry stalks of different humidities and for different internodes.

## 2. Materials and Methods

### 2.1. Method of Preparing Samples for Strength Tests

The plant material used for the research came from experimental plots located at the Experimental Station in Puławy Osinach Institute of Soil Science and Plant Cultivation State Research Institute, Puławy, Poland (51°28′ N and 22°03′ E). Harvesting the miscanthus (Miscanthus × Giganteus) from the experimental plots took place at the turn of February and March.

**Figure 1:**
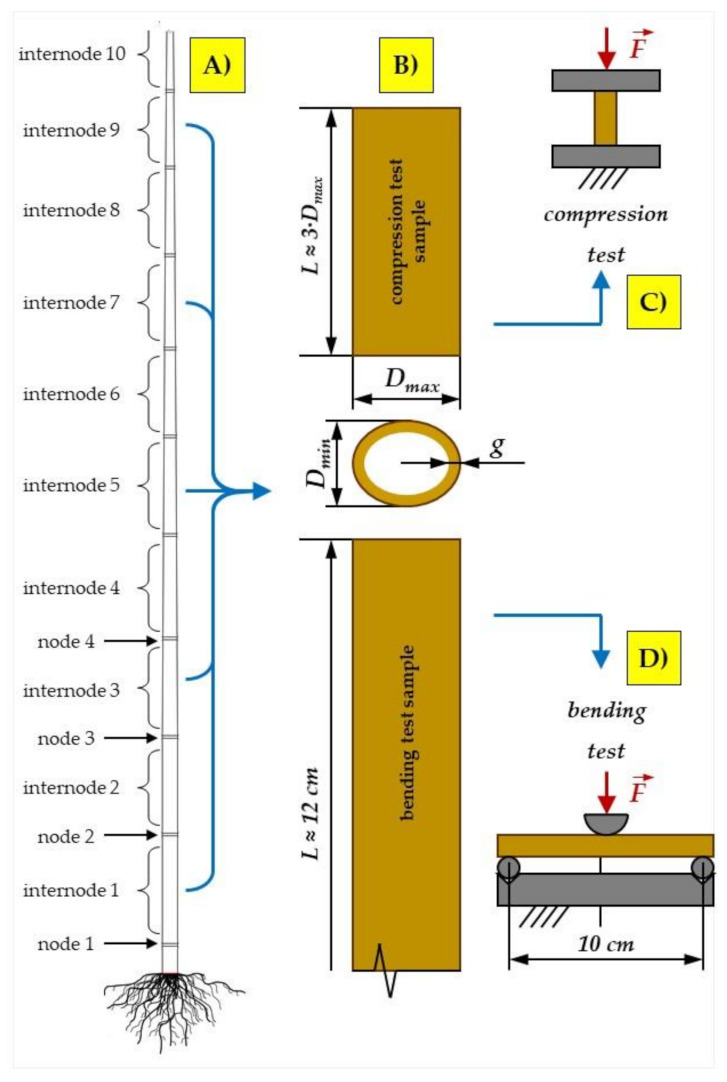
Diagram of preparation and dimensions of samples for compression and bending tests: (**A**) diagram of miscanthus stalk and internode from which samples were taken; (**B**) designation of characteristic dimensions of samples: *D_min_*—minimum stalk diameter, *D_max_*—maximum stalk diameter, *g*—stalk wall thickness; (**C**) diagram of the compression test along the fibers; (**D**) diagram of the three-point bending test.

Samples of 1, 3, 5, 7 and 9 internodes (NrNod) were collected from randomly selected miscanthus stalks (devoid of leaves) (Figure 1A). The samples for the three-point bending tests were cut to a length of about 12 cm (the bending test assumed a support spacing of 10 cm). The samples for compression tests along the fibers were cut to a length approximately equal to three times the diameter of the stalk cross-section (Figure 1B). This allowed us to eliminate the risk of buckling of the sample subjected to compressive load.

To ensure the parallelism of the cross-sections (upper and lower) and their perpendicularity to the stalk axis, the samples for compression tests were cut using the Microm Ergostar HM 200 microtome.

**Figure 2:**
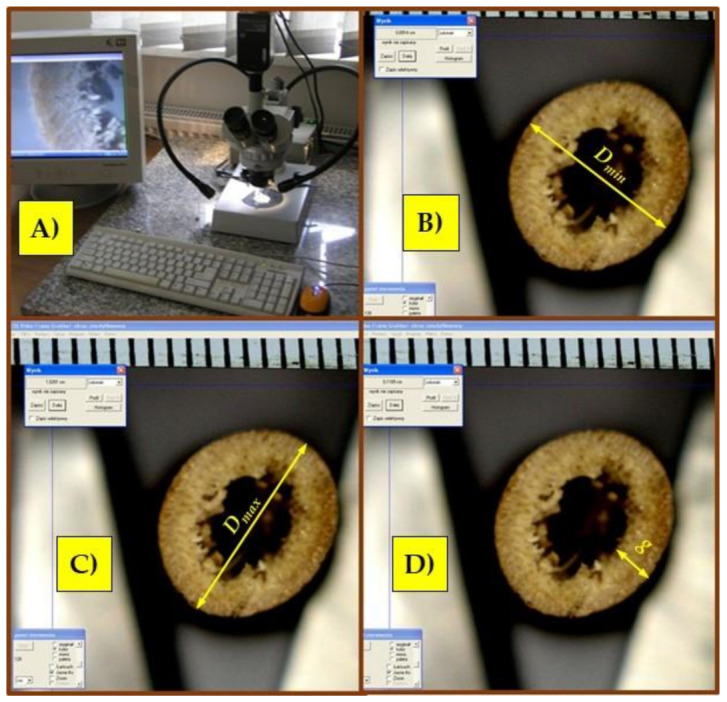
Measurement of the dimensions of the stalk cross-sections for compression and bending tests: (**A**) stand for taking pictures of the cross-sections; (**B**) measurement of the minimum diameter (*D_min_*); (**C**) measurement of the maximum diameter (*D_max_*); (**D**) measurement of the stalk wall thickness (*g*).

A stand equipped with a Hund WETZLAR stereoscopic microscope (Helmut Hund GmbH, Welzlar, Germany) with a built-in Panasonic GP-KR222E digital camera was used to determine the geometric dimensions of the miscanthus stalk cross-section (minimum diameter Dmin, maximum diameter Dmax and wall thickness g—Figure 1B). The recorded image using the DigiLab 4.5 program (DigiLabs Inc., Palo Alto, CA, USA) was generated on a computer screen and recorded in jpg format (Figure 2A). For the obtained photos, the dimensions of the stalk cross-sections were measured using the Multiscan v. 14.96 program (Computer Scanning System, Warsaw, Poland) (Figure 2B–D). Before starting the measurements, the image was scaled by marking two points on the linear gauge scale visible on the image and assigning it a specific length unit. A Stainless Hardened electronic caliper with the accuracy class L = 0.1 mm was used to measure the height L of the samples for compression tests.

### 2.2. Measurement of Moisture Content

As the purpose of the research was to determine the biomechanical parameters of a miscanthus stalk at different moisture contents (MC), it was necessary to determine the MC before each of the strength tests.

The water content of miscanthus stalks was determined with the drying method in accordance with PN EN 13183-1: 2002 [67]. The research material consisted of samples that were collected from each internode of miscanthus stalks. The collected samples were ground and then weighed on a WPS 510/C/1 laboratory scale (Radwag, Radom, Poland) and placed in an ELKON KC 100 KN thermoregulated oven (Zakład Elektroniki i Automatyki Przemysłowej “ELKON” Sp. z o.o., Rybnik, Poland). Drying took place at a temperature of 105 °C. The samples were dried to a constant weight.

The following equation was used to determine the moisture content [42,57,58,60,68,69,70]:

(1) $$\mathrm{MC}=\frac{m_{1}-m_{0}}{m_{0}}\cdot 100\%,$$

where:

MC—moisture content, as a percentage,

$m_{1}$—the mass of the test sample before drying, in kilograms,

$m_{0}$—the mass of the oven-dry test sample, in kilograms.

The determination of the water content was carried out in five repetitions before the individual strength tests were performed.

### 2.3. Static Compression Tests along the Fibres

Static axial compression of the stalks along the fibers was performed on samples from internodes 1, 3, 5, 7 and 9 of miscanthus giganteus. Compression tests were carried out for two levels of water content (MC = 20.7% and MC = 25.8%). The adopted dimensions of the samples (*L* ≈ 3·*D_max_*—Figure 1A) allowed to eliminate the risk of buckling of the material subjected to loading. The assumed measuring length of the samples during the static compression test along the fibers was equal to three stem diameters.

**Figure 3:**
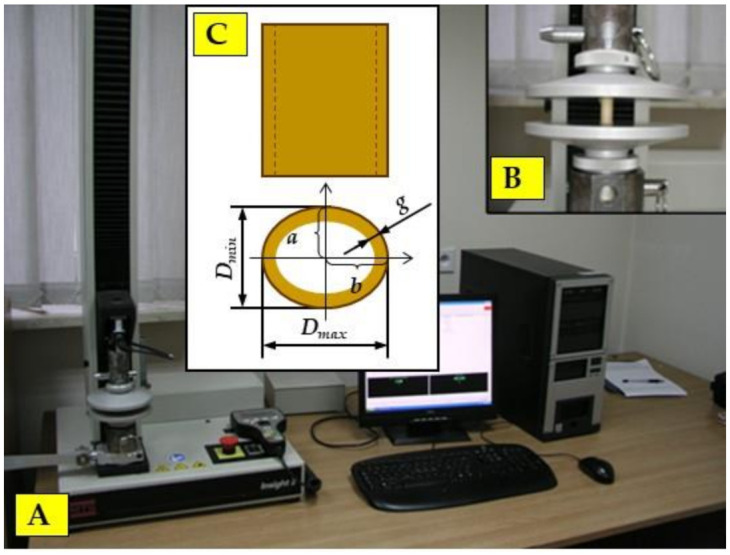
Stand for testing the static compression test: (**A**) MTS testing machine; (**B**) static compression attachment; (**C**) characteristic dimensions of the miscanthus stalk cross-section: *D_min_*—minimum stalk diameter, *D_max_*—maximum stalk diameter, *g*—stalk wall thickness, a—the length of the semi-minor axis of the ellipse, b—the length of the semi-major axis of the ellipse.

The axial compression test was carried out with the use of a special attachment for compressing biological materials installed on the MTS testing machine (MTS Systems Corporation, Eden Prairie, MN, USA), which is shown in Figure 3. The 569327-03 strain gauge head (MTS System Corporation, Eden Prairie, MN, USA)with a measuring range of up to 2 kN was used. The movement speed of the strain gauge head was 4 mm min^−1^.

**Figure 4:**
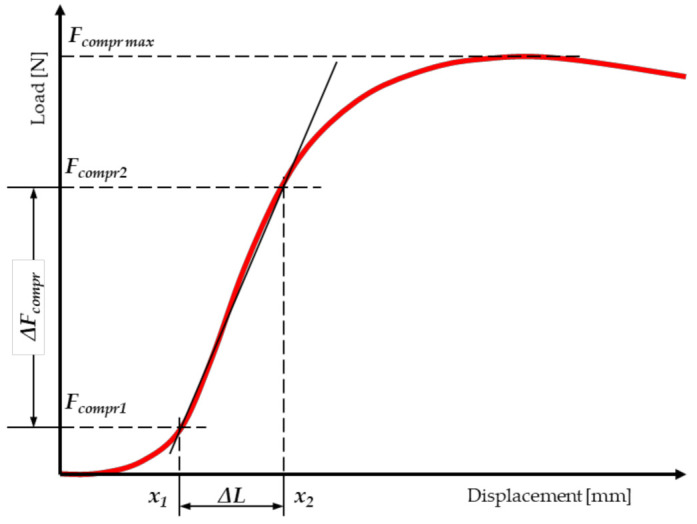
Dependence of force on displacement for the static compression test along the fibers of miscanthus stalk.

It was assumed that the cross-section of miscanthus stalk has the shape of an ellipse, with the ellipse being hollow in the center. The measurement of force and displacement on the MTS testing machine is performed automatically. The program searches the graph (Figure 4) for points that correspond to the initial force (*F_compr1_*) and the basic force (*F_compr2_*), above which the load–displacement relationship (displacement) loses its linear characteristic. In order to determine the modulus of elasticity (MOE_compr_), a mathematical formula is entered into the program.

The values of the modulus of elasticity in compression along the fibers were calculated from the equation [60,69,71,72]:

(2) $${\mathrm{MOE}}_{\mathrm{compr}}=\frac{\sigma }{\varepsilon }=\frac{\Delta F_{compr}}{A_{ellipse}}\cdot \frac{L_{0}}{\Delta L}=\frac{F_{compr2}-F_{compr1}}{\pi \cdot \left(a\cdot b-a_{1}\cdot b_{1}\right)}\cdot \frac{L_{0}}{x_{2}-x_{1}}$$

where:

${\mathrm{MOE}}_{\mathrm{compr}}$—modulus of elasticity in compression along the fibers (MPa),

*σ*—compressive stresses (MPa),

*ε*—relative deformation (-),

$\Delta F_{compr}$—change in the value of the compressive force in the range of the linear characteristic (N),

$A_{ellipse}$—area of elliptical cross-section of the stalk (mm^2^),

*L*_0_—sample initial length (mm),

Δ*L*—displacement within the range of the linear characteristic (mm),

$F_{compr1}$—value of the compressive initial force (N),

$F_{compr2}$—value of the main compressive force (N),

a—the length of the semi-minor axis of the ellipse (mm),

b—the length of the semi-major axis of the ellipse (mm),

$a_{1}$—the semi-minor axis of the ellipse *a* minus the stalk wall thickness *g* (mm),

$b_{1}$—the semi-major axis of the ellipse *b* minus the stalk wall thickness *g* (mm),

$x_{1}$—lower displacement (mm),

$x_{2}$—upper displacement (mm).

The next step was to calculate the load capacity of the stalk, expressed as the maximum stress, corresponding to the destruction of the test sample during the compression test (Figure 4). The value of the bearing capacity was determined from the equation [57,60,65,68,69,70,71,72,73,74]:

(3) $$\sigma_{\mathrm{compr}}=\frac{F_{compr\;max}}{A_{ellipse}}=\frac{F_{compr\;max}}{\pi \cdot \left(a\cdot b-a_{1}\cdot b_{1}\right)}$$

where:

$\sigma_{\mathrm{compr}}$—the maximum stress in compression along the fibers (MPa),

$F_{compr\;max}$—maximum value of the compressive force (N),

$A_{ellipse}$—area of elliptical cross-section of the stalk (mm^2^),

a—the length of the semi-minor axis of the ellipse (mm),

b—the length of the semi-major axis of the ellipse (mm),

$a_{1}$—the semi-minor axis of the ellipse *a* minus the stalk wall thickness *g* (mm),

$b_{1}$—the semi-major axis of the ellipse *b* minus the stalk wall thickness *g* (mm).

The static axial compression tests of the stalks along the fibers were performed in replicates of 15 for each of the five internodes (NrNod = 1, 3, 5, 7 and 9) and for each of the two moisture contents (MC = 20.7% i MC = 25.8%).

### 2.4. Static Bending Tests

**Figure 5:**
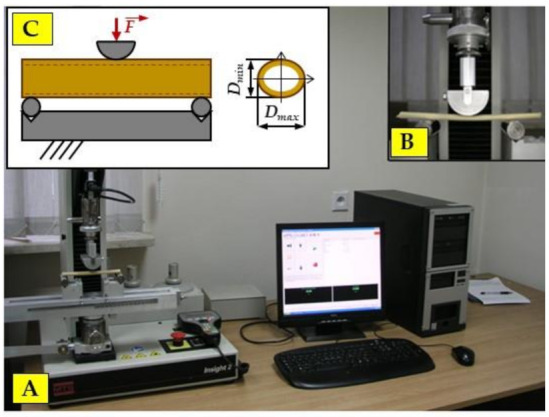
Stand for the three-point bending test: (**A**)—MTS testing machine; (**B**)—static bending attachment; (**C**) three-point bending scheme and characteristic dimensions of the miscanthus stalk cross-section: *D_min_*—minimum stalk diameter, *D_max_*—maximum stalk diameter, *g*—stalk wall thickness, a—the length of the semi-minor axis of the ellipse, b—the length of the semi-major axis of the ellipse.

A static three-point bending test was used to determine the bending strength of a miscanthus stalk. Similar to the compression test, the elliptical shape of the miscanthus stalk cross-section was assumed. The test was carried out with the use of the MTS Insight 2 testing machine (shown in Figure 5), which is the equipment of the laboratory of the Department of Mechanical Engineering and Agrophysics.

During the miscanthus bending test, the support spacing was 100 mm, which was consistent with the methodology of determining the elasticity coefficient for grain stalks. The supports were made in the shape of cylinders with a diameter of 25 mm, the pressing pin had a semicircular shape with a diameter of 60 mm. The movement speed of the strain gauge head was 100 mm min^−1^.

**Figure 6:**
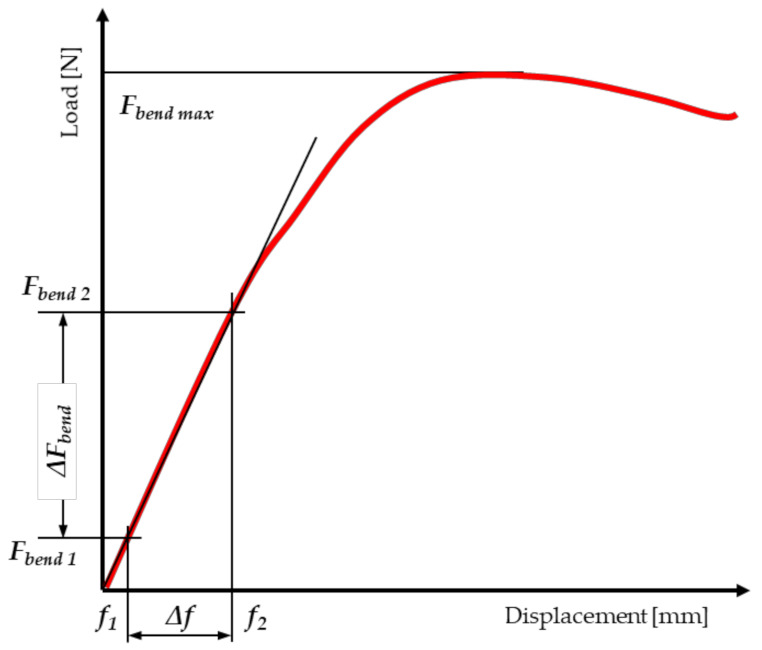
Dependence of force on displacement for the static compression test along the fibers of miscanthus stalk.

The modulus of elasticity for bending (MOEbend) was determined, similarly to compression, using the load–displacement relationship obtained from the MTS testing machine (Figure 6).

The modulus of elasticity for bending miscanthus was calculated from the equation [44,47,52,65,69,75]:

(4) $${\mathrm{MOE}}_{\mathrm{bend}}=\frac{\Delta F_{bend}\cdot L^{3}}{48\cdot I_{major}\cdot \Delta f}=\frac{\left(F_{bend2}-F_{bend1}\right)\cdot L^{3}}{12\cdot \pi \cdot \left(f_{2}-f_{1}\right)\cdot \left(a^{3}\cdot b-a_{1}^{3}\cdot b_{1}^{}\right)}$$

where:

${\mathrm{MOE}}_{\mathrm{bend}}$—modulus of elasticity bending (MPa),

$\Delta F_{bend}$—change in the value of the bending force in the range of the linear characteristic (N),

*L*—distance between supports (mm),

$I_{major}$—moment of inertia around neutral axis (ellipse major axis) (mm^4^),

Δf—the deflection of the stem in the range of the linear characteristic (mm),

$F_{bend1}$—value of the initial bending force (N),

$F_{bend2}$—value of the main bending force (N),

$f_{1}$—initial deflection of the stem (mm),

$f_{2}$—stem deflection for *F_bend_*_2_ (mm),

a—the length of the semi-minor axis of the ellipse (mm),

b—the length of the semi-major axis of the ellipse (mm),

$a_{1}$—the semi-minor axis of the ellipse *a* minus the stalk wall thickness *g* (mm),

$b_{1}$—the semi-major axis of the ellipse *b* minus the stalk wall thickness *g* (mm).

The next step was to calculate the load capacity of the stalk, expressed as the maximum stress, corresponding to the failure of the test specimen using the bending test (Figure 5). The load capacity was calculated from the equation [69,76,77]:

(5) $$\sigma_{bend\;max}=\frac{M_{bendmax}\cdot a}{I_{major}}=\frac{\frac{F_{bend}\cdot L}{4}\cdot a}{\frac{\pi }{4}\cdot \left(a^{3}\cdot b-a_{1}^{3}\cdot b_{1}^{}\right)}=\frac{F_{bend}\cdot L\cdot a}{\pi \cdot \left(a^{3}\cdot b-a_{1}^{3}\cdot b_{1}^{}\right)}$$

where:

*σ_bendmax_*—maximum stress in bending (MPa),

*M_bendmax_*—maximum bending moment about neutral axis (N·mm),

*I_major_*—moment of inertia around neutral axis (ellipse major axis), (mm^4^),

*a*—the perpendicular distance to the neutral axis (mm) (ellipse major axis),

*F_bend_*—maximum bending force value (N),

*L*—distance between supports (mm),

*a*—the length of the semi-minor axis of the ellipse, the perpendicular distance to the neutral axis (mm) (ellipse major axis) (mm),

*b*—the length of the semi-major axis of the ellipse (mm),

*a*_1_—the semi-minor axis of the ellipse *a* minus the stalk wall thickness *g* (mm),

*b*_1_—the semi-major axis of the ellipse *b* minus the stalk wall thickness *g* (mm).

The static three-point bending tests of the stalks were performed in replicates of 15 for each of the five internodes (NrNod = 1, 3, 5, 7 and 9) and for each of the three moisture contents (MC = 22.6%, MC = 29.8%, MC = 38.8%).

### 2.5. Statistical Analysis

All of the quantitative data (MOE_compr_, *σ*_compr_, MOE_bend_, *σ*_bend_,) are presented as the mean value ± 95% confidence interval.

The two-way analysis of variance (ANOVA) was used to check if the analyzed parameters had influence on the variables [78,79,80,81,82]. ANOVA was conducted for each of the following dependent variables: MOE_compr_, *σ*_compr_, MOE_bend_, *σ*_bend_. The internode number (NrNod) and the moisture content (MC), in turn, were the intra-group factors. The internode number (NrNod) was analyzed on 5 levels (1, 3, 5, 7 and 9), while the moisture content (MC) was analyzed on 2 levels in compression along the fibers (20.7%, 25.8%) and on 3 levels in bending (22.6%, 29.8%, 38.8%).

Two-way ANOVA calculations (NrNod, MC factors) were performed in a few steps:

1. Calculating the Sum of Squares (SS), both total and for individual factors:

(6) $$SS_{Total}=SS_{NrNod}+SS_{MC}+SS_{\left(NrNod*MC\right)}+SS_{Error}$$

where:

*SS_Total_*—total sum of squares,

*SS_NrNod_*—inter-group sum of squares for the factor NrNod,

*SS_MC_*—inter-group sum of squares for the factor MC,

SS_(NrNod*MC)_—sum of squares for the interaction of both factors (interaction of the factor NrNod and the factor MC),

*SS_Error_*—within-samples sum of squares.

2. Calculating the degrees of freedom (df):

(7) $$df_{Total}=df_{NrNod}+df_{MC}+df_{\left(NrNod*MC\right)}+df_{Error}$$

(7a) $$\left(n-1\right)=\left(k_{NrNod}-1\right)+\left(k_{MC}-1\right)+\left(k_{NrNod}-1+k_{MC}-1\right)+\left(n-k_{NrNod}-k_{MC}\right)$$

where:

*df_Total_*—the total number of degrees of freedom,

*df_NrNod_*—the number of degrees of freedom for the factor NrNod,

*df_MC_*—the number of degrees of freedom for the factor MC,

SS_(NrNod*MC)_—the number of degrees of freedom for the interaction of factors NrNod and MC,

*SS_Error_*—the number of degrees of freedom for the error,

*n*—total number of measurements,

*k_NrNod_*—number of levels of NrNod factor NrNod (*k_NrNod_* = 5),

*k_MC_*—number of levels of MC (*k_MC_* = 2 for comprresion, *k_MC_* = 3 for bending).

3. Calculating the Mean Squares (MS):

(8) $$\left\{\begin{array}{c} \begin{array}{c} MS_{NrNod}=\frac{SS_{NrNod}}{df_{NrNod}}\\ MS_{MC}=\frac{SS_{MC}}{df_{MC}} \end{array}\\ \begin{array}{c} MS_{\left(NrNod*MC\right)}=\frac{SS_{\left(NrNod*MC\right)}}{df_{\left(NrNod*MC\right)}}\\ MS_{Error}=\frac{SS_{Error}}{df_{Error}} \end{array} \end{array}\right.;$$

where:

*MS_NrNod_*—mean square for the factor NrNod,

*MS_MC_*—mean square for the factor MC,

SS_(NrNod*MC)_—mean square for the interaction of factors NrNod and MC,

*SS_Error_*—mean square for error.

4. Calculating the F-test value:

(9) $$\left\{\begin{array}{c} F_{NrNod\left(k_{NrNod}-1,n-k_{NrNod}-k_{MC}\right)}=\frac{MS_{NrNod}}{MS_{Error}}\\ F_{MC\left(k_{MC}-1,n-k_{NrNod}-k_{MC}\right)}=\frac{MS_{MC}}{MS_{Error}}\\ F_{NrNod*MC\left(k_{NrNod}-1+k_{MC}-1,n-k_{NrNod}-k_{MC}\right)}=\frac{MS_{\left(NrNod*MC\right)}}{MS_{Error}} \end{array}\right.;$$

where:

$F_{NrNod}$—F-test value for factor NrNod,

$F_{MC}$—F-test value for factor MC,

$F_{NrNod*MC}$—F-test value for the interaction of factors NrNod and MC.

5. If the probability p of obtaining the observed value of the F test is less than 0.05, we reject the null hypothesis (H0: all group means are equal) in favor of the alternative hypothesis (H1: at least two group means differ from each other).

If we reject the null hypothesis, we must conduct a more detailed analysis of the differences between the means of individual groups. For this purpose, we use post-hoc tests, which make it possible to determine which group means differ significantly from each other (between which there are no statistically significant differences—you can group the means at the significance level p (define the so-called homogeneous groups, which are marked with subsequent letters of the alphabet). One such test is the HDS test (Honestly Significant Difference Test) developed by Tukey.

In order to determine homogeneous groups (marked on graphs by the same letters), the Honest Significant Difference (HSD) of the Tukey’s test was used [78,80,82].

Data analysis was performed using the STATISTICA software (Dell Inc., (Tulsa, OK, USA, 2016). Dell Statistica (data analysis software system), version 13. software.dell.com). Values of *p* < 0.05 were considered statistically significant.

## 3. Results

### 3.1. The Results of Samples’ Geometrical Dimension Measurements for Strength Tests

Table 2 and Table 3 summarize the measured cross-sectional dimensions of miscanthus stalk samples used in the compression along the grain and three-point bending tests. As the internode number increases, the values of *D_max_*, *D_min_* and g decrease significantly.

With the increase in the internode number, the mean values of the diameters *D_max_* decreased from 9.8 mm to 6.7 mm and *D_min_* from 8.8 mm to 5.5 mm, while the wall thickness g decreased the most from 1.5 mm to 0.7 mm.

### 3.2. Strength Test Results

In Table 4 the measurement results obtained in the compression strength tests along the fibers of miscanthus giganteus and three-point bending tests are summarized. The average values and standard deviations of elasticity modulus and maximum stresses for individual internodes and different moisture contents are included.

The results of the analysis of variance are presented in Table 5, Table 6, Table 7, Table 8.

**Figure 7:**
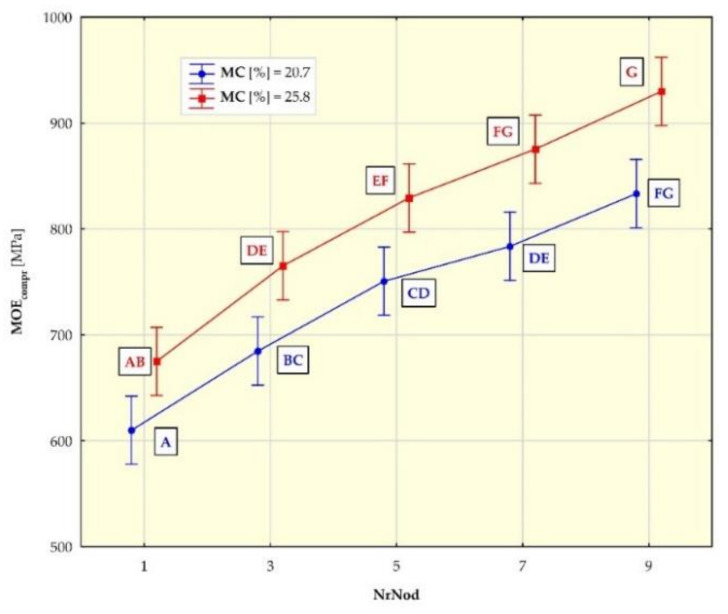
Average modulus of elasticity in compression (MOE_comp_) values for different internodes (NrNod) and moisture content (MC). All data are expressed as mean ± 95% confidence interval (the latter being represented by whiskers in the diagram). Different letters indicate significant differences between the groups according to Tukey’s test (*p* < 0.05). Homogeneous groups are marked with the same letters.

**Figure 8:**
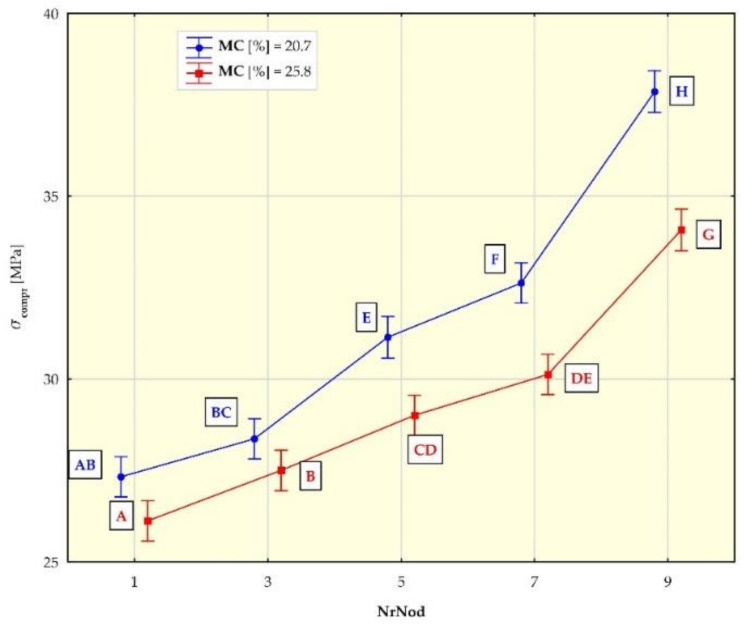
Average maximum stress in compression (σ_comp_) values for different internodes (NrNod) and moisture content (MC). All data are expressed as mean ± 95% confidence interval (the latter being represented by whiskers in the diagram). Different letters indicate significant differences between the groups according to Tukey’s test (*p* < 0.05). Homogeneous groups are marked with the same letters.

**Figure 9:**
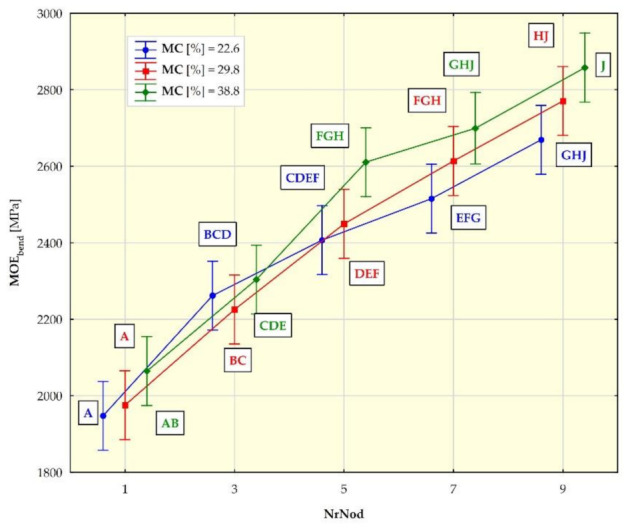
Average modulus of elasticity in bending (MOE_bend_) values for different internodes (NrNod) and moisture content (MC). All data are expressed as mean ± 95% confidence interval (the latter being represented by whiskers in the diagram). Different letters indicate significant differences between the groups according to Tukey’s test (*p* < 0.05). Homogeneous groups are marked with the same letters.

**Figure 10:**
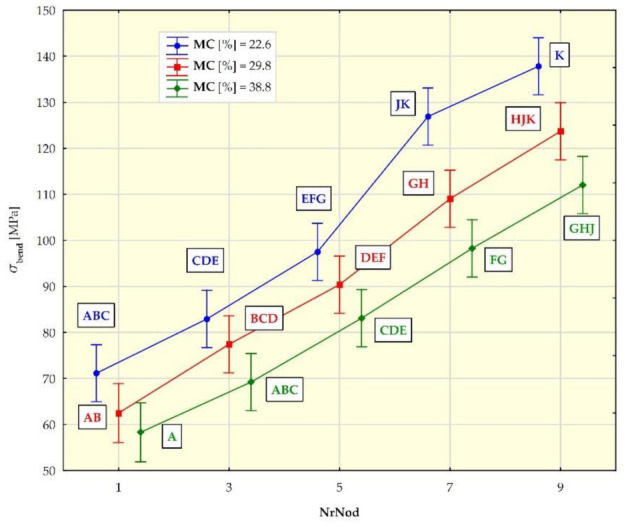
Average maximum stress in bending (σ_bend_) values for different internodes (NrNod) and moisture content (MC). All data are expressed as mean ± 95% confidence interval (the latter being represented by whiskers in the diagram). Different letters indicate significant differences between the groups according to Tukey’s test (*p* < 0.05). Homogeneous groups are marked with the same letters.

Figure 7, Figure 8, Figure 9 and Figure 10 show the mean values of the biomechanical parameters (modulus of elasticity in compression MOE_compr_, maximum stress in compression σ_compr_, modulus of elasticity in bending MOE_bend_ and maximum stress in bending σ_bend_) for the individual values of the independent variables (NrNod and MC).

Individual homogeneous groups, determined on the basis of the HSD Tukey test, to which individual averages belong, are marked with capital letters. For example, for MOEcompr (Figure 7), the homogeneous group A consists of means for NrNod = 1 and MC = 20.7% as well as NrNod = 1 and MC = 25.8%. Homogeneous group B consists of means for NrNod = 1 and MC = 25.8% as well as NrNod = 3 and MC = 20.7%.

## 4. Discussion

The mean values of MOE_compr_ for MC = 20.7% for individual internodes are respectively (Table 4): 610 MPa, 685 MPa, 751 MPa, 784 MPa, 833 MPa. Whereas for MC = 25.8% the mean values of MOE_compr_ are: 675 MPa, 765 MPa, 829 MPa, 875 MPa, 930 MPa.

The mean values of σ_comp_ for MC = 20.7% are (Table 4): 27.3 MPa, 28.4 MPa, 31.1 MPa, 32.6 MPa, 37.9 MPa (respectively for the 1-3-5-7-9 internode). For MC = 25.8% the mean values of σ_comp_ are: 26.1 MPa, 27.5 MPa, 29.0 MPa, 30.1 MPa, 34.1 MPa (for the 1-3-5-7-9 internode).

No compression test results along the grain for miscanthus were found in the available literature. The results of our research can be compared with those obtained by Jimenez-Espada et al. [69] in compression tests along the fibers of a reed stem with a moisture of 15%. The average values of MOE_compr_ amounted to 4200 MPa, and the ultimate stress in compression was 52 MPa.

The mean values obtained in our research are significantly lower and amount to 31.5 MPa for σ_compr_, and to 733 MPa for MOE_compr_, for similar humidity (MC = 20.7%).

For MOE_bend_, the mean values amount to (Table 4): 1948 MPa, 2262 MPa, 2407 MPa, 2515 MPa, 2669 MPa (for the 1-3-5-7-9 internode) for MC = 22.6%. For MC = 29.8% mean values of MOE_bend_ are: 1976 MPa, 2226 MPa, 2449 MPa, 2613 MPa, 2771 MPa and for MC = 38.8% mean values are: 2065 MPa, 2304 MPa, 2611 MPa, 2699 MPa, 2858 MPa.

For the maximum bending stress (σ_bend_) the mean values for individual internodes 1-3-5-7-9 (Table 4) amount to, respectively: 71.1 MPa, 82.9 MPa, 97.5 MPa, 126.9 MPa, 137.8 MPa (for MC = 22.6%). For higher water contents, the mean values σ_bend_ are lower: 62.5 MPa, 77.4 MPa, 90.4 MPa, 109.0 MPa, 123.7 MPa (for MC = 29.8%) and 58.3 MPa, 69.2 MPa, 83.1 MPa, 98.3 MPa, 112.0 MPa (for MC = 38.8%).

The comparison of the mean values of MOE_bend_ and σ_bend_ (obtained in the three-point bending tests) with the values obtained for compression of the miscanthus stalk along the fibers shows that they are significantly higher. For a similar water content (MC = 22.6%) the mean MOE_bend_ values are 2360 MPa (3.3 times greater) and σ_bend_ 103.2 MPa (3.2 times greater).

Our average MOE_bend_ results can also be compared with data obtained by other researchers. Słupska et al. [44] obtained the average value of MOE_bend_ = 3440 MPa, Kaack and Schwarz [48] MOE_bend_ = 4260 MPa and Kaack et al. [49] MOE_bend_ = 2567 MPa. These values are comparable to those obtained by us. On the other hand, Liu et al. [40] obtained a significantly higher value of the mean MOE_bend_ = 8443 MPa. Dunn and Dabney [54] for the miscanthus sinensis stalk determined an average MOE_bend_ of 4500 MPa.

Only Słupska et al. [44] determined the maximum bending stress and obtained the mean value of σ_bend_ = 41.6 MPa, more than two times lower than what was obtained in our tests.

The ANOVA results (Table 2, Table 3, Table 4 and Table 5) show a statistically significant effect of the independent variables—the moisture content (MC) and the internode number (NrNod)—on all four dependent variables: modulus of elasticity in compression (MOE_compr_), maximum stress in compression (σ_comp_), modulus of elasticity in bending (MOE_bend_) and maximum stress in bending (σ_bend_). Only for the maximum stress in compression (σ_comp_) variable, there is a statistically significant interaction of the MC * NrNod factors.

The values of the modulus of elasticity in compression (MOE_compr_) (Figure 7) increase with the increasing number of the internode. For individual internodes, MOE_compr_ values are higher for higher moisture contents in the tested material. The mean values of the maximum compressive stress σ_comp_ (Figure 8) increase with the increasing number of the internode. For individual internodes, they are lower for higher moisture contents (MC). The results of our research show that the values of the biomechanical parameters (MOE_compr_ and σ_comp_) obtained in the compression tests along the miscanthus stalk fibers increase with the increase in the internode number. As MC increases, the MOE_compr_ values increase, while the σ_comp_ values decrease.

Unfortunately, in the available literature, there are no other miscanthus test results concerning MOE_compr_ and σ_comp_ with which the results obtained by us could be compared. However, it is possible to compare the tendencies of changes obtained by us to the results of the research on bamboo shoots, which also belong to the grass family Poaceae.

**Figure 11:**
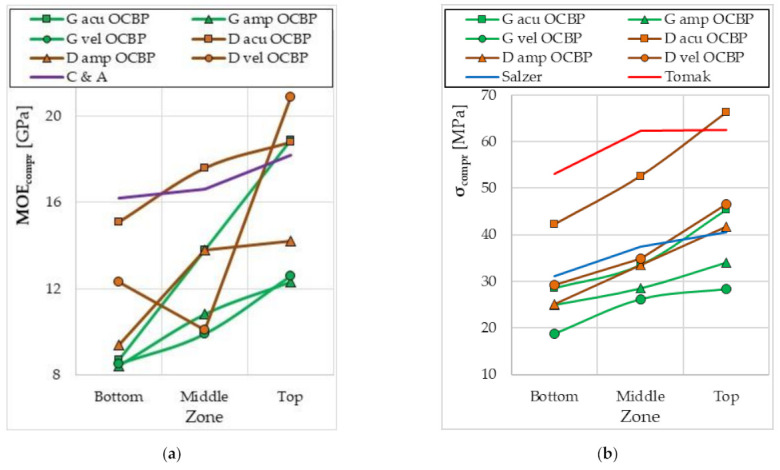
Changes in the values of biomechanical parameters depending on the bamboo shoot zone obtained in compression tests along the fiber: (**a**) modulus of elasticity in compression MOEcompr; (**b**) maximum stress in compression σcompr. G acu—Guadua aculeata bamboo in a green state; G amp—Guadua amplexifolia bamboo in a green state; G vel—Guadua velutina bamboo in green state; D acu—Guadua aculeata bamboo in a dry state; D amp—Guadua amplexifolia bamboo in a dry state; D vel OCBP—Guadua velutina bamboo in a dry state; OCBP—Ordonez-Candelaria and Bárcenas-Pazos 2014 [60]; C and A—Correal and Juliana Arbeláez 2010 [83]; Salzer—Salzer et al., 2018 [57]; Tomak—Tomak et al., 2012 [59].

The results of several studies on the influence of the internode (shoot height) from which the samples were taken on the values of MOE_compr_ and σ_comp_ were found in the literature (Figure 11) [57,59,60,83]. In these studies, no specific internode number was identified, but the lower, middle and upper parts of the bamboo shoot were examined. It can be concluded that the values tend to increase the MOE_compr_ value with the increase in the inter-node number (Figure 11a). Additionally, in the case of the maximum compressive stress, the tendency is similar—there is an increase in σ_comp_ with an increase in NrNod (Figure 11b). Thus, the trends in the changes of the MOE_compr_ and σ_comp_ values obtained for the research on bamboo shoots are the same as what were obtained in our research.

The mean values of modulus of elasticity in bending (MOE_bend_) obtained by us in the three-point bending tests of the miscanthus giganteus (Figure 9) increase with the increase in the internode number (the tendency is similar to that in the case of MOE_compr_). In the case of bending, the MOE_bend_ values are higher for a higher water content only for internodes 1, 5, 7 and 9, and the value differences are not as pronounced as in the case of compression (value ranges coincide). The mean values of the maximum bending stress (σ_bend_) obtained by us (Figure 10) increase with the increase in the internode number (similar to compression). For individual internodes, the values of σ_bend_ are lower for higher moisture contents (MC).

Thus, the results of our research show that the values of the biomechanical parameters (MOE_bend_ and σ_bend_) obtained in the miscanthus three-point bending tests increase with the increase in the internode number. For individual internodes, the MOE bend values are slightly higher for higher moisture contents. On the other hand, the values of σ bend are smaller for a higher MC. The trends are therefore similar to compression along the fibers.

**Figure 12:**
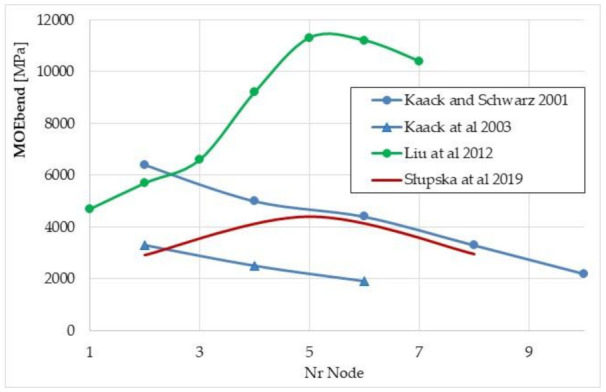
Changes in the value of modulus of elasticity in bending MOE_bend_ depending on the number of the internode for the giant miscanthus: Kaack and Schwarz 2001 [48], Kaack et al., 2003 [49], Liu et al., 2012 [40], Słupska et al., 2019 [44].

The results published by Liu et al. [40] indicate a similar trend of changes in the value of the bending modulus as in our research—an increase in the MOE_bend_ value with an increase in the internode number (Figure 12).

According to the research by Słupska et al. [44], the MOE_bend_ value initially increases with increasing NrNod and then decreases. The tests were carried out for three zones (basal, middle and apical zones), not for specific internodes, and for different humidities (25%, 16% and 14%, respectively). On the other hand, the results obtained by Kaack and Schwarz and Kaack et al. (Kaack and Schwarz 2001, Kaack et al., 2003) indicate the opposite tendency—the MOE_bend_ value decreases with an increase in the internode number (Figure 12).

There are no results in the literature concerning the changes of the σ_bend_ value depending on the internod e number.

**Figure 13:**
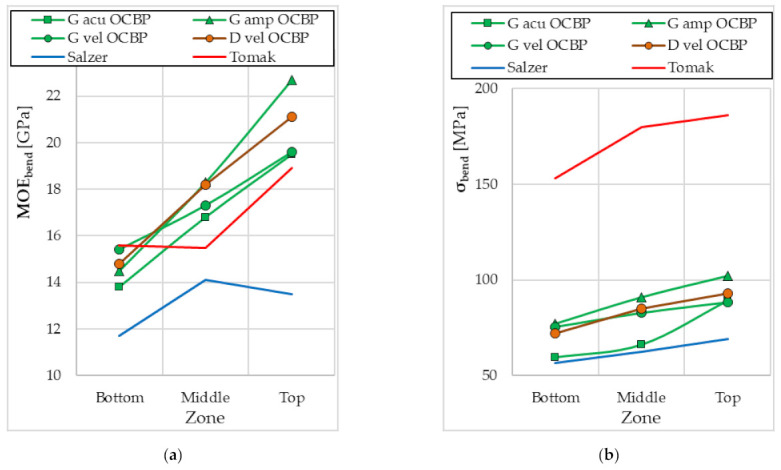
Changes in the values of biomechanical parameters depending on the bamboo shoot zone obtained in the three-point bending tests: (**a**) modulus of elasticity in bending MOEbend; (**b**) maximum stress in bending σbend. G acu—Guadua aculeata bamboo in a green state; G amp—Guadua amplexifolia bamboo in a green state; G vel—Guadua velutina bamboo in green state; D vel—Guadua velutina bamboo in a dry state; OCBP—Ordonez-Candelaria and Bárcenas-Pazos 2014 [60]; Salzer—Salzer et al. 2018 [57]; Tomak—Tomak et al., 2012 [59].

The results obtained for bamboo shoots (Figure 13) may serve as an additional confirmation of the tendencies of changes in MOE_bend_ and σ_bend_ obtained in our research.

According to the results of studies of various bamboo species published by Ordonez-Candelaria and Bárcenas-Pazos [60], the MOE_bend_ values increase linearly with an increase in the internode number (bottom, middle and top zones) (Figure 13a). Similar upward trends were obtained by Salzer et al. [57] and Tomak et al. [59].

Clear upward trends were also obtained for σ_bend_ (Figure 13b). The values obtained by Tomak et al. [59] are significantly larger than those obtained by other research teams.

Differences in the values of the biomechanical parameters (modulus of elasticity in compression, maximum stress in compression, modulus of elasticity in bending and maximum stress in bending) may result from several reasons. These parameters depend, inter alia, on the material moisture—only in some tests is the water content in miscanthus stalks given, though it differs from the material moisture in our tests. Another factor that may affect the values of elasticity modules and maximum stresses may be habitat conditions (soil composition, fertilization, irrigation) and climatic conditions. There are different clones within the species Miscanthus × Gigantheus, which may differ in their biomechanical properties. Such a large diversity of the results obtained by different authors may also result from the different developmental phases of the studied plants.

Differences in the obtained values of elasticity modules and maximum stresses are also influenced by the differences in the calculation models adopted by the researchers. This especially applies to the method of calculating the moment of inertia for circular stems. Most researchers assumed a circular cross-section of the stem, while in our research an elliptical cross-section was assumed.

## 5. Conclusions

The results obtained in this study proved that the independent variables of the moisture content and the internode number play a statistically significant influence on the values of the examined biomechanical parameters of the miscanthus stem: modulus of elasticity in compression, maximum stress in compression, modulus of elasticity in bending and maximum stress in bending.

The values of the modulus of elasticity (MOE) increase when increasing the internode number. For individual internodes, MOE values are higher for higher moisture contents in the tested material.

The values of the maximum stress (σ) also increase when increasing the internode number. On the other hand, for individual internodes, the σ values are lower for higher moisture contents.

The average values of the modulus of elasticity obtained are in the range from 610 MPa to 930 MPa (for compression) and from 1948 MPa to 2858 MPa (for bending). On the other hand, the average values of the maximum stress range from 26.1 MPa to 37.9 (for compression along the fibers) and from 58.3 MPa to 137.8 MPa (for bending).

## Figures and Tables

**Table 1:** Summary of test results on miscanthus bending (Dry Internodes).

Authors	MOE_bend_ [GPa]	Source
Słupska et al.	2.93–4.42	[44]
Nowakowski et al.	1.70–1.84	[44]
Kaack and Schwartz	2–8 (mean 4.5)	[48]
Kaack et al.	4.27	[49]
Liu et al.	4.6–10.3	[40]

**Table 2:** Dimensions of the miscanthus stalk samples’ cross-section used for compression tests along the grain.

MC ^1^	NrNod ^1^	*D_max_* ^1^ Mean (Max/Min) [mm]	*D_min_* ^1^ Mean (Max/Min) [mm]	*g* ^1^ Mean (Max/Min) [mm]
20.7%	1	9.8 (11.2/9.09)	8.81 (8.91/8.65)	1.46 (1.59/1.37)
	3	8.57 (10.05/7.67)	8 (8.41/7.48)	1.3 (1.42/1.22)
	5	8.35 (9.97/7.3)	6.76 (7.22/5.92)	1.14 (1.24/1.07)
	7	6.99 (8.6/5.75)	5.63 (6.34/4.55)	0.93 (1.01/0.87)
	9	6.68 (8.39/5.36)	5.53 (6.46/4.1)	0.74 (0.8/0.69)
25.8%	1	9.78 (10.66/9.25)	8.83 (8.99/8.63)	1.47 (1.54/1.32)
	3	8.63 (9.54/8)	8.09 (8.57/7.53)	1.31 (1.37/1.18)
	5	8.43 (9.43/7.78)	6.85 (7.38/6.26)	1.15 (1.2/1.03)
	7	6.99 (8.16/6.36)	5.81 (6.47/5.13)	0.94 (0.98/0.84)
	9	6.68 (8.01/6.12)	5.73 (6.4/5.06)	0.74 (0.78/0.67)

^1^ MC—moisture content (%); NrNod—internode number, *D_max_* maximum diameter, *D_min_*—minimum diameter; *g*—stalk wall thickness (*g*).

**Table 3:** Dimensions of the miscanthus stalk samples’ cross-section used for three-point bending tests along the grain.

MC ^1^	NrNod ^1^	*Dmax* ^1^ Mean (Max/Min) [mm]	*Dmin* ^1^ Mean (Max/Min) [mm]	*g* ^1^ Mean (Max/Min) [mm]
22.6%	1	9.6 (10.83/8.27)	8.6 (9.2/8)	1.47 (1.7/1.28)
	3	8.5 (9.83/7.27)	7.91 (8.6/7.3)	1.34 (1.63/1.25)
	5	8.32 (9.99/6.89)	6.77 (7.7/6.1)	1.2 (1.41/1.04)
	7	7.1 (8.71/5.7)	5.67 (6.6/4.8)	1.01 (1.21/0.81)
	9	6.69 (7.95/5.54)	5.55 (6.6/4.7)	0.8 (0.99/0.66)
29.8%	1	9.69 (10.16/9.05)	8.79 (8.9/8.58)	1.46 (1.66/1.12)
	3	8.64 (9.98/7.71)	8.07 (8.56/7.55)	1.29 (1.47/0.99)
	5	8.32 (9.62/7.3)	6.87 (8.19/5.91)	1.15 (1.31/0.88)
	7	6.96 (8.16/5.75)	5.84 (7.08/4.92)	0.94 (1.07/0.72)
	9	6.61 (7.96/5.36)	5.78 (6.94/4.76)	0.75 (0.86/0.58)
38.8%	1	9.66 (10.33/9.05)	8.78 (8.91/8.59)	1.46 (1.59/1.31)
	3	8.63 (9.67/7.77)	8.09 (8.65/7.65)	1.32 (1.44/1.18)
	5	8.42 (9.71/7.66)	6.72 (7.79/6.02)	1.17 (1.27/1.05)
	7	6.98 (8.54/5.98)	5.6 (6.83/4.66)	0.97 (1.05/0.87)
	9	6.66 (8.21/5.74)	5.52 (6.75/4.5)	0.78 (0.85/0.7)

^1^ MC—moisture content (%); NrNod—internode number, *D_max_* maximum diameter, *D_min_*—minimum diameter; *g*—stalk wall thickness (*g*).

**Table 4:** Values of strength parameters of miscanthus stalk (MOE_compr_, σ_compr_, MOE_bend_, σ_bend_) obtained in the compression tests along the fibers and three-point bending test. The mean and standard deviation (SD) values are included.

		Mean (Standard Deviation) [Mpa]				
Parameter	MC [%]	NrNod = 1	NrNod = 3	NrNod = 5	NrNod = 7	NrNod = 9
**MOE_compr_ ^1^** [Mpa]	20.7	610 (85)	685 (50)	751 (91)	784 (39)	833 (61)
	25.8	675 (63)	765 (59)	829 (51)	875 (59)	930 (55)
**σ_compr_ ^1^** [Mpa]	20.7	27.3 (0.4)	28.4 (0.3)	31.1 (0.9)	32.6 (1.3)	37.9 (1.4)
	25.8	26.1 (1.1)	27.5 (1)	29 (1.2)	30.1 (1.3)	34.1 (1.2)
**MOE_bend_ ^1^** [Mpa]	22.6	1948 (257)	2262 (180)	2407 (219)	2515 (236)	2669 (233)
	29.8	1976 (183)	2226 (92)	2450 (193)	2614 (225)	2771 (79)
	38.8	2065 (169)	2304 (99)	2611 (142)	2699 (106)	2858 (79)
**σ_bend_ ^1^** [Mpa]	22.6	71.1 (4.2)	82.9 (5)	97.5 (6.8)	126.9 (13.4)	137.8 (14.5)
	29.8	62.5 (3.3)	77.4 (9.6)	90.4 (15.1)	109 (22)	123.7 (27.4)
	38.8	58.3 (1.8)	69.2 (3.9)	83.1 (4.6)	98.3 (7)	112 (8.7)

^1^ MC—moisture content (%); NrNod—internode number; **MOE_comp_**—modulus of elasticity in compression; σ_comp_—maximum stress in compression; MOE_bend_—modulus of elasticity in bending; σ_bend_—maximum stress in bending.

**Table 5:** Two-way ANOVA results for modulus of elasticity in compression (MOE_compr_).

Source of Variation	SS ^1^	df ^1^	MS ^1^	F ^1^	p ^1^	Significant
MC	255,205	1	255,205	64.13	0.0000	Yes
NrNod	1,038,906	4	259,727	65.27	0.0000	Yes
MC * NrNod	4499	4	1125	0.28	0.8888	No
Error	557,135	140	3980			

^1^ SS—sum of squares between groups, df—number of the degree of freedom, MS—mean sum of squares between groups, F—value of the test statistic, p—probability.

**Table 6:** Two-way ANOVA results for maximum stress in compression (σ_comp_).

Source of Variation	SS ^1^	df ^1^	MS ^1^	F ^1^	p ^1^	Significant
MC	161.4	1	161.4	138.5	0.0000	Yes
NrNod	1487.9	4	372.0	319.1	0.0000	Yes
MC * NrNod	38.4	4	9.6	8.2	0.0000	Yes
Error	159.7	137	1.2			

^1^ SS—sum of squares between groups, df—number of the degree of freedom, MS—mean sum of squares between groups, F—value of the test statistic, p—probability.

**Table 7:** Two-way ANOVA results for modulus of elasticity in bending (MOE_bend_).

Source of Variation	SS ^1^	df ^1^	MS ^1^	F ^1^	p ^1^	Significant
MC	8.408 × 10^5^	2	4.204 × 10^5^	13.42	0.0000	Yes
NrNod	1.636 × 10^7^	4	4.090 × 10^6^	130.59	0.0000	Yes
MC * NrNod	1.796 × 10^5^	8	2.245 × 10^4^	0.72	0.6768	No
Error	6.547 × 10^6^	209	3.132 × 10^4^			

^1^ SS—sum of squares between groups, df—number of the degree of freedom, MS—mean sum of squares between groups, F—value of the test statistic, p—probability.

**Table 8:** Two-way ANOVA results for maximum stress in bending (σ_bend_).

Source of Variation	SS ^1^	df ^1^	MS ^1^	F ^1^	p ^1^	Significant
MC	13,613	2	6806	45.84	0.0000	Yes
NrNod	108,566	4	27,141	182.80	0.0000	Yes
MC * NrNod	1825	8	228	1.54	0.1462	No
Error	30,883	208	148			

^1^ SS—sum of squares between groups, df—number of the degree of freedom, MS—mean sum of squares between groups, F—value of the test statistic, p—probability.

## Data Availability

The data presented in this study are available within the article.
